# Erzhi Pill® Repairs Experimental Liver Injury via TSC/mTOR Signaling Pathway Inhibiting Excessive Apoptosis

**DOI:** 10.1155/2017/5653643

**Published:** 2017-05-30

**Authors:** Bu-gao Zhou, Hai-mei Zhao, Xiu-yun Lu, Xin Wang, Yong Zou, Rong Xu, Hai-yang Yue, Yi Liu, Zheng-yun Zuo, Duan-yong Liu

**Affiliations:** ^1^Science and Technology College, Jiangxi University of Traditional Chinese Medicine, Nanchang, Jiangxi Province 330004, China; ^2^School of Basic Medical Sciences, Jiangxi University of Traditional Chinese Medicine, Nanchang, Jiangxi Province 330004, China; ^3^Department of Postgraduate, Jiangxi University of Traditional Chinese Medicine, Nanchang, Jiangxi Province 330004, China; ^4^Affiliated Hospital, Science and Technology College, Jiangxi University of Traditional Chinese Medicine, Nanchang, Jiangxi Province 330006, China

## Abstract

The present study aimed to investigate the mechanism of hepatoprotective effect of Erzhi Pill (EZP) on the liver injury via observing TSC/mTOR signaling pathway activation. The experimental liver injury was induced by 2-acetylaminofluorene (2-AAF) treatment combined with partial hepatectomy (PH). EZP treated 2-AAF/PH-induced liver injury by the therapeutic and prophylactic administration. After the administration of EZP, the activities of aspartic transaminase (AST), alanine aminotransferase (ALT), alkaline phosphatase (AKP), and gamma-glutamyl transpeptidase (*γ*-GT) were decreased, followed by the decreased levels of hepatocyte apoptosis and caspase-3 expression. However, the secretion of albumin, liver weight, and index of liver weight were elevated. Microscopic examination showed that EZP restored pathological liver injury. Meanwhile, Rheb and mammalian target of rapamycin (mTOR) activation were suppressed, and tuberous sclerosis complex (TSC) expression was elevated in liver tissues induced by 2-AAF/PHx and accompanied with lower-expression of Bax, Notch1, p70S6K, and 4E-EIF and upregulated levels of Bcl-2 and Cyclin D. Hepatoprotective effect of EZP was possibly realized via inhibiting TSC/mTOR signaling pathway to suppress excessive apoptosis of hepatocyte.

## 1. Introduction

Liver injury can be induced by different stimuli such as alcohol intake, viral infection, cholestasis, steatosis, and drug abuse [1–5]. In the damaged liver, cell death modes include apoptosis, necrosis, necroptosis, and autophagy. Apoptosis is an early, chronic, and temperate response subsequent to injury induction, whereas necrosis is an acute and severe response [6–8]. It is known that apoptosis is a prominent pathological feature in all liver diseases [9, 10]. Excessive hepatocyte apoptosis is bound to induce hypohepatia in damaged liver. Therefore, apoptosis not only is important in the pathophysiology of human liver disease but also is on the edge of entering clinical practice by providing new treatment opportunities. Increasing evidences have shown that the inhibition of hepatic apoptosis can reverse or delay the progression of liver diseases, as demonstrated by preclinical models and clinical trials. So, inhibition of hepatic apoptosis is a significant therapeutic option for liver injury and an important method of drug efficacy evaluation [6–8].

Mammalian target of rapamycin (mTOR) is classically recognized as the central kinase that controls cell proliferation and translation, which is a well-known 289 kDa PI3-kinase present in all eukaryotic organisms [11, 12]. In recent years, the mTOR pathway regulates a variety of cell functions, including cell proliferation, survival, growth, apoptosis, metabolism, and plasticity. Tuberous sclerosis complex (TSC) negatively regulates activation of mTOR pathway via Rheb, which is an upstream protein of mTOR signaling pathway and including TSC1 and TSC2. The Rheb is required for activation of mTOR towards examining all substrates and leading to many human diseases [13].

An increasing number of evidences have indicated that human diseases have been closely correlated with TSC/mTOR pathway dysregulation, including many types of cancer, cardiovascular diseases, hepatic disease, immunological disorders, and neurological disorders [14, 15]. Intriguingly, hyperactivity of TSC/mTOR pathway plays a significant role in most of these diseases as hepatic disease. Experimental and clinical trials had revealed that, as a representative of mTOR inhibitors, Rapamycin (RAPA) has the therapeutic potential for treatment of immune-mediated liver injury in the clinic, which is an effective immunosuppressant widely used for preventing immune activation. Many previous studies showed that RAPA inhibited apoptosis to treat many diseases via suppressing TSC/mTOR signaling pathway to regulate the expression levels of apoptosis-related proteins as caspase-3, caspase-9, and Bax, and proinflammatory cytokines as IFN-gamma and TNF-alpha [16, 17]. According to the above analysis, we think that TSC/mTOR pathway inhibition is an attractive therapeutic option of hepatic disease which might modify the process of apoptosis.

As a famous Chinese medicine formula, Erzhi Pill (EZP) is used to treat and prevent various chronic hepatic diseases, such as chronic hepatitis, alcoholic hepatitis, alcoholic cirrhosis, and toxic cirrhosis [18, 19]. EZP is composed of* Fructus Ligustri Lucidi *and* Herba Ecliptae* (Table 1) and serves as a basic formula to effectively treat hepatic diseases [18, 19]. An increasing number of studies have explored the pharmacology of EZP and herbs composed of EZP, showing their antioxidant, anti-inflammatory, and antiaging properties and potential in immunoregulation, liver protection, antinerve cell apoptosis, and cell proliferation (Table 1) [20–28]. At present, most researches are focused on evaluating the therapeutic effect of EZP on hepatic diseases by liver function examination in animal or cell models with acute hepatic injury induced by carbon tetrachloride (CCl4), H2O2, and ethanol [29–31]. Available data have shown that EZP and its active ingredients significantly decreased the activity of serum alanine aminotransferase (ALT) and aspirate aminotransferase (AST), decreased malondialdehyde (MDA) content in liver, and increased superoxide dismutase (SOD) activity [29–31]. However, insufficient evidence is available to prove that the therapeutic and prophylactic administration of EZP prevents hepatic diseases by inhibiting hepatic apoptosis via TSC/mTOR signaling pathway.

## 2. Material and Methods

### 2.1. Animals

Male Wistar rats weighing 180–220 g were originally obtained from the Animal Center of Peking University Health Science Center (the animal certificate number was SCXK 2006-0008). These animals were in-house bred and maintained on standard laboratory chow and daily 12-hour light/dark cycles. The rats were maintained under constant room temperature (25°C) and provided with free access to a standard diet and water in accordance with institutional guidelines. The rats were acclimatized to these conditions for 7 days prior to any involvement in experimental studies. All animal experiments were conducted according to the protocols (JZ2014-216) approved by the Animal Care and Use Committee of Jiangxi University of Traditional Chinese Medicine (TCM). The rats were randomly distributed into five groups: normal (Normal), 2-acetylaminofluorene (2-AAF)/partial hepatectomy (PH) model (Model), 2-AAF/PH rats prevented with EZP (EZPP), 2-AAF/PH rats treated with EZP (EZPT), and 2-AAF/PH rats treated with Rapamycin (RAPA). Each group comprised eight rats.

### 2.2. Drugs

EZP (batch number Z32020882) was produced by Lei Yun Shang Pharmaceutical Company (Shanghai, China). Rapamycin (RAPA, batch number 20150483) was purchased from Wyeth Pharmaceuticals Company (PA, USA). 2-AAF was from Sigma (MO, USA).

### 2.3. Induction by 2-Acetylaminofluorene/Partial Hepatectomy

All surgical procedures were performed under sodium pentobarbital anesthesia. Except for rats in the Normal group, the 2-AAF/PH model (2-AAF/PHx) was induced in rats according to the procedure described by Shiota and his colleagues [32, 33]. Briefly, 15 mg/kg of 2-AAF was administered daily by gavage for 7 days. A standard one-third PH was performed 7 days after starting the 2-AAF administration. Experimental procedures were approved by the Animal Care Committee of Jiangxi University of TCM and performed in compliance with the guidelines of the university. The animals received human care according to the criteria outlined in the* Guide for the Care and Use of Laboratory Animals* prepared by the National Academy of Sciences. For the Normal group, the abdomens of rats were opened, and 1 mL of whole blood was taken from the portal vein.

### 2.4. Pharmacological Treatments

When 2-AAF was administered in the Model and EZPP groups, 6.48 g/kg of EZP was orally administered to rats until they were sacrificed. After PHx in the EZPT and RAPA groups, all rats were given 6.48 g/kg of EZP or 1 mg/kg of Rapamycin for 14 days. Meanwhile, the rats in the Normal and Model groups were orally given the same volume of physiological saline until they were sacrificed. After anesthesia with 10% urethane, the rats were sacrificed on the fifteenth day after PH.

### 2.5. Hematoxylin and Eosin Staining

After the rats (*n* = 8) were sacrificed, the liver obtained from each animal was fixed in 4% paraformaldehyde, embedded in paraffin, sectioned at 8 *μ*m thickness, and stained with hematoxylin and eosin (HE). Histological observations were made by light microscopy.

### 2.6. Assessment of Liver Functions

Peripheral blood (*n* = 8) was collected from the aorta ventralis in rats anesthetized with urethane and separated into serum. The serum was analyzed using automatic biochemical analyzer. The main indexes of liver functions included albumin, ALT, AST, gamma-glutamyl transpeptidase (*γ*-GT), and alkaline phosphatase (AKP).

### 2.7. Hepatocyte Isolation

Hepatocytes were isolated by two-step collagenase perfusion of the liver followed by isodensity centrifugation in Percoll as described [34]. Viability was determined by trypan blue exclusion and was >90%.

### 2.8. Assessment of Liver Apoptosis and Caspase-3 Protein

Cell apoptosis was analyzed using the Annexin V-fluorescein isothiocyanate (FITC)/propidium iodide (PI) according to the instructions of the Apoptosis Kit (FITC, BD Pharmingen, CA, USA) and the previous study [35]. Briefly, the cells were seeded in 100 mm culture dishes. After being washed twice with NaCl/PI, the cells (*n* = 8) were resuspended in binding buffer and incubated with FITC-conjugated Annexin V for 10 min in the dark at room temperature. Then, the resuspended cells were incubated with PI solution for 30 min in the dark at 37°C.

Other cells (*n* = 8) were fixed in Fix/Perm Buffer (eBioscience, CA, USA) for at least 1 h at 37°C and then incubated with cleaved caspase-3 antibody (FITC, BD Pharmingen) for 1 h in the dark at 37°C. All stained cells were tested by flow cytometry (Becton Dickinson, San Jose, CA, USA). The cells labeled with FITC rat IgG2a were used as the isotype negative control. Per sample, 100,000 events were acquired. The data were analyzed using the FlowJo software (Becton Dickinson, San Jose, CA, USA).

### 2.9. Western Blot Analysis

Protein concentrations (*n* = 6) were determined in the supernatant of liver tissues by classic BCA protein assay (Beyotime). Equal protein of each sample was fractionated onto sodium dodecyl sulfate polyacrylamide gel electrophoresis (SDS-PAGE) and transferred onto polyvinylidene fluoride (PVDF) membrane by a Bio-Rad Western blot apparatus. The membranes were blocked with 5% fat-free milk or 5% bovine serum albumin and then probed with the following primary antibodies for 12 hours at 4°C: GAPDH (1 : 2000), Anti-Bax (1 : 2000), Anti-Bcl-2 (1 : 1000), Anti-Rheb (1 : 2000), Anti-Tuberin (1 : 1000), Anti-p-Tuberin (1 : 500), Anti-mTOR (1 : 800), Anti-p-mTOR (1 : 800), Anti-Notch1 (1 : 1000), Anti-Cyclin D (1 : 1000), Anti-p70S6K (1 : 1000), and Anti-4E-EIF (1 : 1000) (Abcam, Cambridge, UK). The membranes were incubated with appropriate horseradish peroxidase-conjugated secondary antibodies (1 : 2000~1 : 3000, Abcam, Cambridge, UK) and visualized with an enhanced chemiluminescence (ECL) detection kit (Millipore). Bands were quantified using quantity one 4.40 software (Bio-Rad, CA, USA).

### 2.10. Statistical Analysis

Data were expressed as mean ± standard error of the mean (SEM). The statistical significance was determined using one-way analysis of variance followed by the Tukey-Kramer post hoc test and performed by Prism 4.0 (GraphPad Software, CA, USA). A *p* value < 0.05 was considered significant.

## 3. Results

### 3.1. EZP Attenuated Pathological Injury in Rats with 2-AAF/PH-Induced Liver Injury

**Figure 1:**
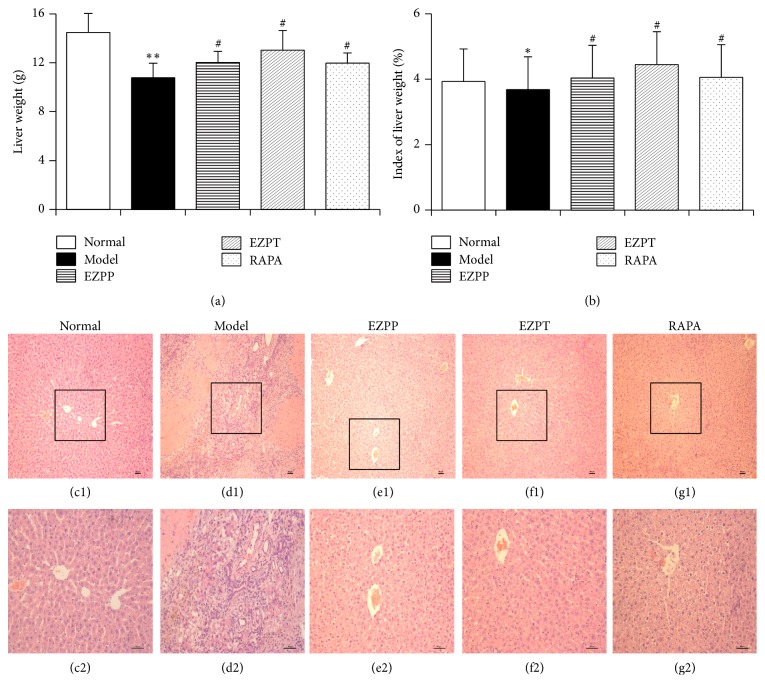
*Pathological changes in liver injury induced by 2-AAF/PH*. The index of liver weight (a) was measured as liver weight (g)/body weight (g) × 100%. (a): liver weight; (b): index of liver weight; liver histology is shown in Normal ((c1) and (c2)), Model group ((d1) and (d2)), EZPP group ((e1) and (e2)), EZPT group ((f1) and (f2)), and RAPA group ((g1) and (g2)); pathology slides of liver were stained with hematoxylin and eosin. (c1), (d1), (e1), (f1), and (g1): Bar = 100 *μ*m; (c2), (d2), (e2), (f2), and (g2): Bar = 200 *μ*m. Data were expressed as mean ± standard error of the mean (*n* = 8). ^*∗*^*p* < 0.05 and ^*∗∗*^*p* < 0.01 versus Normal group; ^#^*p* < 0.05 versus Model group.

Liver histology was studied in the 2-AAF/PH rats and other rats by H&E staining. Although hepatocytes have vigorous vitality, 2AAF-induced disorder of hepatocyte regeneration limited the self-repair ability of liver, hence aggravating liver injury.  Figure 1 shows the structural disorder of hepatic lobules, mass hepatocyte necrosis, hepatocyte fatty degeneration, physaliphora formation, inflammatory cell infiltration, and tissue edema followed by diseased liver weight (Figure 1(a)) and index of liver weight (Figure 1(b)) in the liver tissue section from rats in the 2-AAF/PH model (Figures 1(d1) and 1(d2)). However, in the EZPP (Figures 1(e1) and 1(e2)), EZPT group (Figures 1(f1) and 1(f2)), and RAPA group (Figures 1(g1) and 1(g2)), microscopic observation showed central vein hyperemia, fewer hepatocyte necrosis, several hepatocyte fatty degeneration and/or edema, and irregular newborn hepatocytes as dikaryotic or unequal-sized liver cells. The structure of hepatic lobules was intact as before in 2-AAF/PH rats prevented by EZPP and Rapamycin. These results indicated that the prophylactic and therapeutic administration of EZP can attenuate the pathological 2-AAF/PH-induced injury of liver in rats.

### 3.2. EZP Ameliorated Liver Function in Rats with 2-AAF/PH-Induced Liver Injury

**Figure 2:**
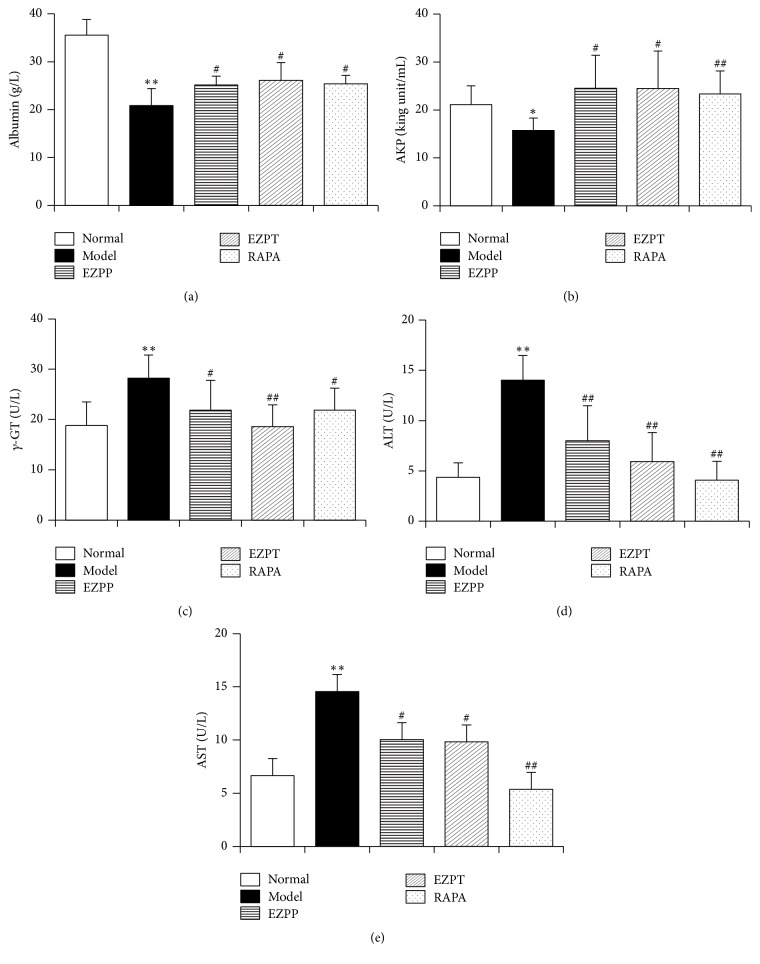
*Liver function detection*. Biochemical parameters in blood related to liver function in rats given EZP after 2-AAF treatment combined with PH. These parameters included albumin (a), AKP (b), *γ*-GT (c), ALT (d), and AST (e). Data were expressed as mean ± standard error of the mean (*n* = 8). ^*∗*^*p* < 0.05 and ^*∗∗*^*p* < 0.01 versus Normal group; ^#^*p* < 0.05 and ^##^*p* < 0.01 versus Model group.

Because of 2-AAF-limited hepatocyte cytothesis and PH, liver damage occurred persistently to induce the dysfunction of hepatic cells on albumin synthesis and deactivation of various enzymes. In the 2AAF/PH model, the secretion of albumin (Figure 2(a)) was decreased, while the levels of AKP (Figure 2(b)), *γ*-GT (Figure 2(c)), ALT (Figure 2(d)), and AST (Figure 2(e)) increased compared with the Normal group. However, compared with the 2-AAF/PH Model group, the yield of albumin (Figure 2(a)) was increased, while the levels of AKP (Figure 2(b)), *γ*-GT (Figure 2(c)), ALT (Figure 2(d)), and AST (Figure 2(e)) were decreased in the EZPP, EZPT, and RAPA group. These results showed that EZP recovered hepatic function to prevent 2-AAF/PH-induced liver injury.

### 3.3. EZP Suppressed Excessive Hepatocyte Apoptosis in Rats with 2-AAF/PH-Induced Liver Injury

**Figure 3:**
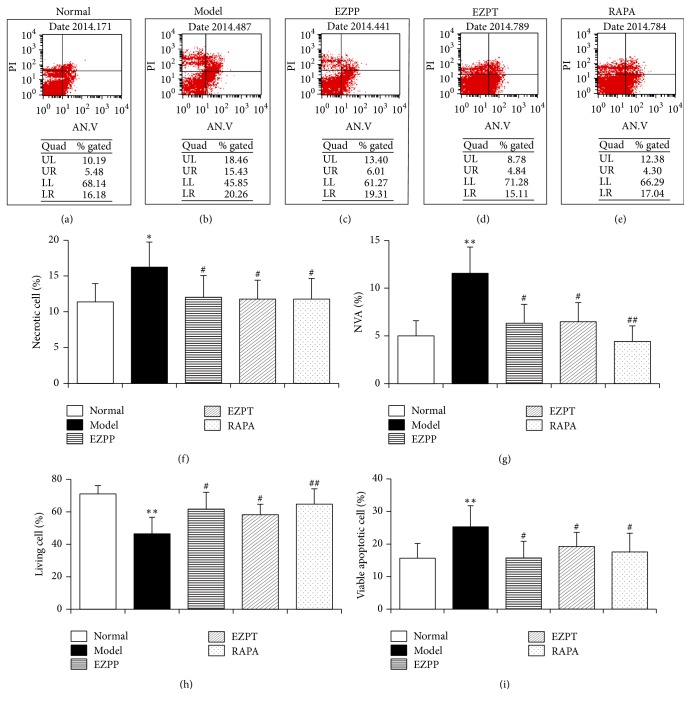
*Hepatocyte apoptosis in liver tissues*. The typical pseudocolors of hepatocyte apoptosis of liver cells were selected from rats in Normal group (a), Model group (b), EZPP group (c), EZPP group (d), and RAPA group (e). The numbers of necrotic cells (NC) (f), nonviable apoptotic cell (NVA) (g), living cells (h), and viable apoptotic cell (VAC) (i) of liver tissues were analyzed by flow cytometry. Data were expressed as mean ± standard error of the mean (*n* = 8). ^*∗*^*p* < 0.05 and ^*∗∗*^*p* < 0.01 versus Normal group; ^#^*p* < 0.05 and ^##^*p* < 0.01 versus Model group.

Hepatocyte apoptosis was an important index to evaluate the levels of liver injury and the capacities of liver restoration. Compared with the Normal group, the numbers of necrotic cell (NC) (Figures 3(a), 3(b), 3(c), 3(d), 3(e), and 3(f)), nonviable apoptotic cell (NVA) (Figures 3(a), 3(b), 3(c), 3(d), 3(e), and 3(g)), and viable apoptotic cell (VAC) (Figures 3(a), 3(b), 3(c), 3(d), 3(e), and 3(i)) were increased in the 2-AAF/PH-treated rats without treatment, while the number of living cells (Figures 3(a), 3(b), 3(c), 3(d), 3(e), and 3(h)) was decreased. However, these tendencies of VAC, NVA, NC, and living cells were contrary to those in the 2-AAF/PH-treated rats which were prophylactically and therapeutically given EZP or Rapamycin (Figure 3). The results indicated that EZP suppressed hepatocyte apoptosis in rats with 2-AAF/PH liver.

### 3.4. EZP Inhibited Caspase-3 Expression of Hepatocytes in Rats with 2-AAF/PH-Induced Liver Injury

**Figure 4:**
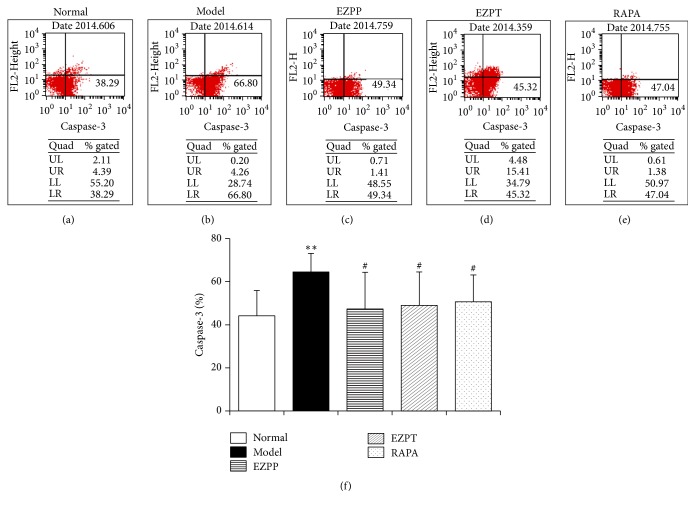
*Caspase-3 expression in liver tissues*. The typical pseudocolors of caspase-3 expression of liver cells were selected from rats in Normal group (a), Model group (b), EZPP group (c), EZPP group (d), and RAPA group (e). Data of caspase-3 expression (f) were expressed as mean ± standard error of the mean (*n* = 8). ^*∗∗*^*p* < 0.01 versus Normal group; ^#^*p* < 0.05 versus Model group.

The caspase-3 is one of the classic indicators of VAC. In the early phase of apoptosis, caspase-3 was usually overexpressed in the 2-AAF/PH rats without treatment (Figure 4). In the present study, caspase-3 expression in liver cells decreased in the EZPP, EZPT, and RAPA groups compared with rats with the 2-AAF/PH-induced liver injury (Figure 4). The result had hinted that EZP inhibited early apoptosis of hepatocytes via caspase-3.

### 3.5. EZP Regulated Rheb and Tuberin Expression in Liver Tissues of Rats with 2-AAF/PH-Induced Liver Injury

**Figure 5:**
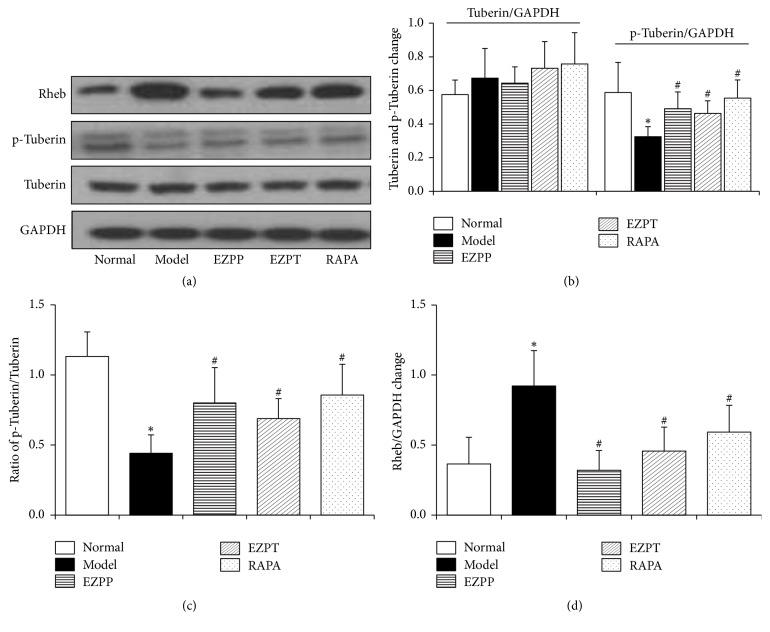
*Western blot analysis of Rheb, Tuberin, and p-Tuberin*. (a) Western blot of Rheb, Tuberin, and p-Tuberin. (b) Quantitative analysis of Tuberin and p-Tuberin. (c) Ratio of p-Tuberin/Tuberin. (d) Quantitative analysis of Rheb. Data are presented as mean ± SEM (*n* = 6). ^*∗*^*p* < 0.05 versus the Normal group; ^#^*p* < 0.05 versus the Model group.

Rheb and Tuberin are two important proteins of mTOR signaling pathway. In the present study, compared with normal rats, the expression of p-Tuberin was signally inhibited in liver tissues of 2-AAF/PH-induced liver injury rats without treatment (Figures 5(a) and 5(b)), which was kept with the same change with the ratio of p-Tuberin/Tuberin (Figure 5(c)). Meanwhile, p-Tuberin expression and ratio of p-Tuberin/Tuberin were elevated in the EZPP, EZPT, and RAPA groups when they were in contrast to the liver injury rats without treatment (Figures 5(a), 5(b), and 5(c)).  Figure 5 showed that the expression of Rheb protein in liver injury rats without treatment was observably lower than rats in the EZPP, EZPT, and RAPA groups (Figures 5(a) and 5(d)). The above results show that EZP inhibited activation of the upstream proteins in TSC/mTOR signaling pathway in 2-AAF/PH-induced liver injury.

### 3.6. EZP Suppressed mTOR Activation in Liver Tissues of Rats with 2-AAF/PH-Induced Liver Injury

**Figure 6:**
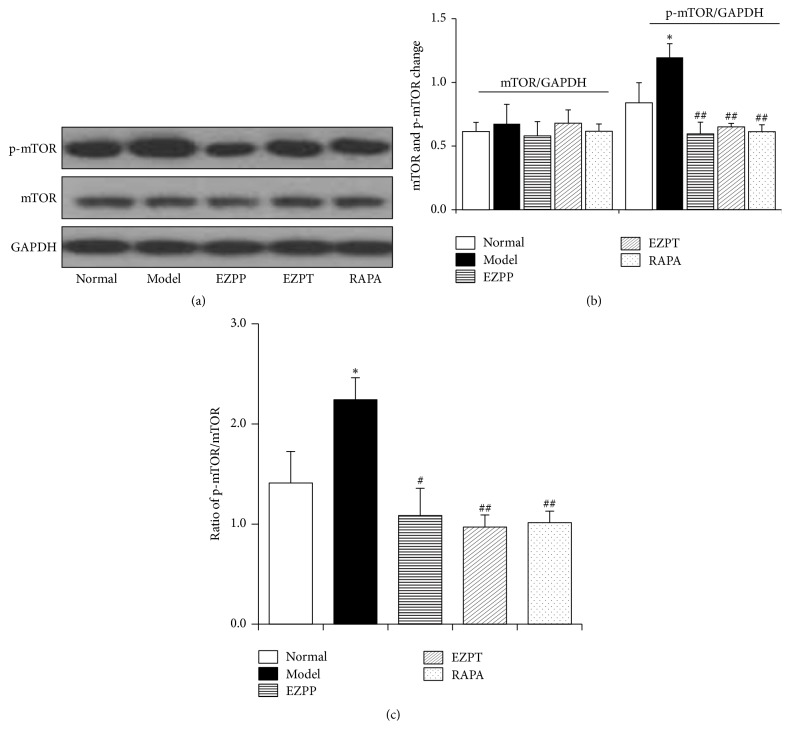
*Western blot analysis of mTOR and p-mTOR*. (a) Western blot of mTOR and p-mTOR. (b) Quantitative analysis of mTOR and p-mTOR. (c) Ratio of p-mTOR/mTOR. Data are presented as mean ± SEM (*n* = 6). ^*∗*^*p* < 0.05 versus Normal group; ^#^*p* < 0.05 and ^##^*p* < 0.01 versus Model group.

mTOR protein is a pivotal molecule which can activate cascade response of apoptosis. Compared with rats the Normal group, the extent of mTOR activation was heightened in liver tissues of the 2-AAF/PH-treated rats in the Model group. However, the p-mTOR expression was suppressed when the 2-AAF/PH-treated rats were treated by therapeutic and prophylactic administration of EZP or Rapamycin (Figures 6(a), 6(b), and 6(c)). These changes had indicated that EZP inhibited the activation of mTOR in liver tissues of the 2-AAF/PH-treated rats.

### 3.7. EZP Inhibited Expression of Downstream Proteins Regulated TSC/mTOR Pathway

**Figure 7:**
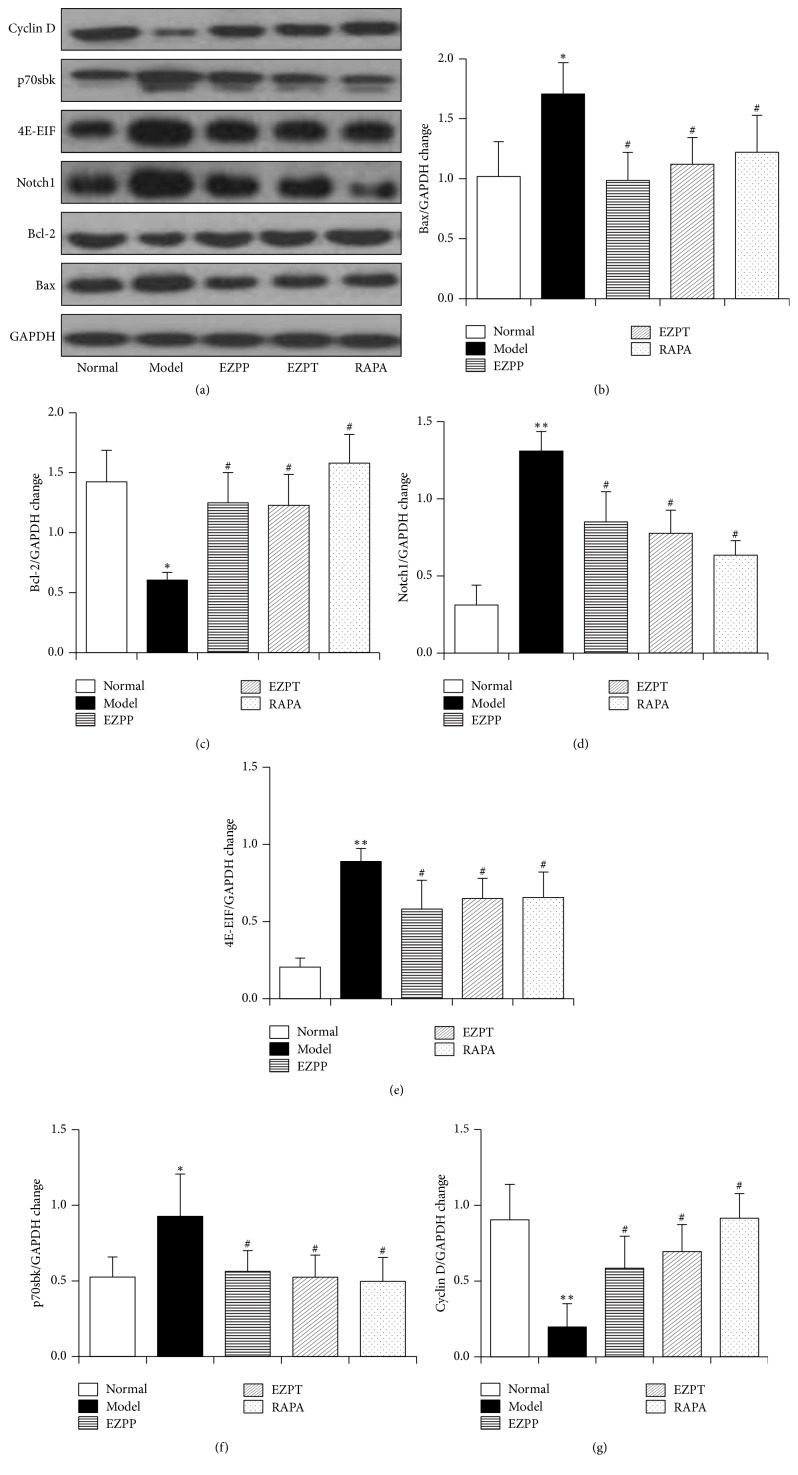
*Western blot analysis of Rheb, Tuberin, and p-Tuberin*. (a) Western blot of Bax, Bcl-2, Notch1, 4E-EIF, p70S6K, and Cyclin D. (b) Quantitative analysis of Bax. (c) Quantitative analysis of Bcl-2. (d) Quantitative analysis of Notch1. (e) Quantitative analysis of 4E-EIF. (f) Quantitative analysis of p70S6K. (g) Quantitative analysis of Cyclin D. Data are presented as mean ± SEM (*n* = 6). ^*∗*^*p* < 0.05 and ^*∗∗*^*p* < 0.01 versus the Normal group; ^#^*p* < 0.05 versus the Model group.

We analyzed that the downstream proteins regulated TSC/mTOR pathway are including Bax, Bcl-2, Notch1, Cyclin D, p70S6K, and 4E-EIF, which are closely regulated apoptosis proteins. In Figure 7, the proteins of Bax, Notch1, p70S6K, and 4E-EIF were highly expressed in liver tissues of the 2-AAF/PH-treated rats without administration, when they were compared with the normal rats (Figures 7(a), 7(b), 7(d), 7(e), and 7(f)), while yields of these proteins of rats in the EZPP, EZPT, and RAPA groups were lower than these in the Model groups (Figures 7(a), 7(b), 7(d), 7(e), and 7(f)). However, the Cyclin and Bcl-2 protein were upregulated in the EZPT, EZPT, and RAPA groups when they were compared with the Model group (Figures 7(a), 7(c), and 7(g)). These results hinted that EZP inhibited apoptosis-regulated proteins which are downstream proteins of TSC/mTOR signaling pathway.

## 4. Discussion

Mature hepatocytes have a vivifying replication capability to effectively restore the structure of hepatic parenchyma after liver injury caused by a variety of methods such as partial hepatectomy (PHx), hepatic toxins, and hepatotropic virus infection. However, factors such as 2-AAF and CCL4 induce hepatocyte injury to limit the regenerative ability of liver. 2-AAF may possess a mechanism that is analogous to a wide variety of hepatotoxins [33]. An exposure to 2-AAF can lead to hepatocyte death and destruction of cellular structures and functions in animals. Previous studies have illustrated that the 2-AAF/PH animal model of liver injury is one of the most widely used models of liver diseases to explore the mechanism of action and therapeutic effect of various medicines [36, 37].

In the present study, liver pathological slices of 2-AAF/PH-treated rats without treatment showed many pathological changes including destroyed hepatic lobules, steatosis, hepatocyte necrosis, and inflammatory cell infiltration at day 14 post-PH. Meanwhile, the levels of serum enzymes (ALT, AST, AKP, and *γ*-GT) increased; similar was the effect on hepatocyte apoptosis, as the numbers of VAC, NVA, and necrotic cells and the expression of caspase-3 increased. These results had shown that liver injury was successfully induced by 2-AAF treatment combined with PH, followed by excessive apoptosis in liver tissues. However, after therapeutic and prophylactic administration of EZP or Rapamycin, all changes were reversed and ameliorated to recover the structures of hepatic lobules and liver function. It hinted that EZP and Rapamycin effectively treated and prevented 2-AAF/PHx-induced liver injury.

Mammalian target of rapamycin (mTOR) is a serine/threonine kinase that modulates several important aspects of mammalian cell function which included the initiation of protein translation, cell proliferation, mortality, and survival protein synthesis [38, 39]. As an upstream protein of mTOR signaling pathway, tuberous sclerosis complex (TSC) plays an important role in the regulation of cell proliferation and differentiation processes through negative mTOR pathway regulation. TSC is composed of TSC1 (locus 9q34) or TSC2 (locus 16p13.3) [40, 41], which, respectively, encode the proteins Hamartin and Tuberin [42]. Higher level or activation of Tuberin, which is a product of the tumor suppressor gene TSC-2 and inhibits Rheb, can inhibit activation of the mTOR signaling pathway [43–45].

The canonical pathway of TSC/mTOR activation by trophic factors starts with the activation of downstream proteins, having a variety of functions, including autophagy, apoptosis, cell proliferation, survival, growth, metabolism, and plasticity [46, 47]. Especially on apoptosis, mTOR may also have a pleiotropic function in the regulation of apoptosis [48]. Recently increasing evidences showed that TSC/mTOR pathway plays a significant role in the activation of this apoptotic signaling cascade. Kato et al. had indicated that mTOR was shown to be activated in ER stress-induced apoptosis as well as in cadmium-induced cell death, which was related to mTOR-IRE1-JNK pathway, MAPK/mTOR network [49, 50]. The regulation of TSC/mTOR signaling pathway on apoptosis was dictated by the cellular context and by multiple downstream targets including apoptosis-regulatory proteins such Cyclin D, eIF4E, p70S6K, p53, Bad, and Bcl-2. Apoptotic signals are activated through activation of the mTOR pathway [48]. On the one hand, mTOR phosphorylates and activates p70S6K (p70 ribosomal protein S6 kinase), furthermore, to involve in phosphorylation/inactivation of Bcl-2 [51, 52]. As is well-known, Bcl-2 is a negative regulator of apoptosis. Phosphorylation of Bcl-2 inactivates its antiapoptotic effect and triggers the release of cytochrome c, then activating caspase-3 which induced early apoptosis [53, 54]. And then mTOR can activate eIF4E, which is associated with upregulation of expression of proapoptotic protein Bax [55]. On the other hand, Notch signaling is a developmental pathway that regulates several fundamental cellular processes including cell fate and differentiation [56, 57]. A positive regulation of Notch by mTOR accelerates cell cycle in the regulation of apoptosis via activating Cyclin D to induce apoptosis [58]. Velagapudi and his colleagues had indicated that blocking mTOR activity is an important pathway involved in inactivation of the apoptosis cascade signals. Inhibition of mTOR activation by rapamycin significantly decreases apoptosis. Their study hinted that phosphorylation/activation of the mTOR, substrate p70S6K, inactivation of Bcl-2, and activation of caspase-3 led to apoptosis of tubular epithelial cells in the kidney cortex of rats [48]. In the present study, after 2AAF/PH-treated liver injury in rats, the key proteins of mTOR signaling pathway, including Rheb and p-mTOR, were activated, but Tuberin was inhibited. And furthermore the apoptosis-regulated proteins (including Bax, Notch1, p70S6K, and 4E-EIF) were high-expressed. The results indicated that activation of TSC/mTOR signaling pathway was the significant character in the pathogenesis of 2-AAF/PHx-induced liver injury. The inhibition function of TSC was decreased to activate Rheb and then heighten mTOR expression. High-expressed mTOR finally induced excessive liver apoptosis via activating Bax, Notch1, p70S6K, and 4E-EIF, or downregulating Bcl-2, or inhibiting Cyclin D to accelerate cell cycle. These results illustrated that TSC/mTOR pathway activation played a critical role in pathogenesis of 2-AAF/PHx-induced liver injury.

Intriguingly, hyperactivity of mTOR pathway was usually found in the process of many hepatic diseases in clinical and experimental cases. In CCl4-induced hepatic fibrosis in mice without treatment, the level of mTOR phosphorylation was elevated and participated in hepatic fibrogenesis, followed by high-expressed p-p70S6K, while luteolin ameliorates liver fibrosis via suppressing AKT/mTOR/p70S6K signaling pathway [59]. Many previous studies found that the levels of pAKT and p-mTOR were significantly higher in liver tissues infected by hepatitis virus B and hepatitis virus C. Zhu and his colleagues found that p-AKT and p-mTOR expressions were enhanced 7 day after transfection and increased for 28 days, meanwhile firstly indicating that HBx-induced alpha-fetoprotein (AFP) expression critically promotes malignant transformation in liver cells through the activation of PI3K/mTOR signaling [60]. Bose et al. observed that an increased level of Rheb, p70S6K level, and mTOR expression, accompanied with a decreased expression of TSC1/TSC2 in HCV-infected hepatocytes, furthermore, indicated that HCV core protein plays a significant role in modulating the mTOR/S6K1 signaling pathway [61]. The similar results were found in the many studies on the hepatitis, hepatocellular carcinoma, fatty liver, hepatic malignancies, and liver transplantation [62–65]. Abundant studies indicated that activation of TSC/mTOR signaling pathway is a common and effective direction to explore the pathogenesis and therapeutic strategy of liver injury. And furthermore, it is well-known that excessive hepatocyte apoptosis is a prominent pathological feature in all liver diseases and bound to induce hypohepatia in damaged liver [9, 10]. So we think that dysregulation of the TSC/mTOR pathway on apoptosis is emerging as a common theme in diverse hepatic diseases; it is hinted that target TSC/mTOR pathway is a feasible therapeutic strategy to treat hepatic diseases.

As a prototype drug of mTOR pathway inhibitors, Rapamycin (RAPA) effectively treated liver injury induced by concanavalin A (Con A), realizing that RAPA reduced transaminase levels and suppressed proinflammatory cytokines IFN-gamma and TNF-alpha production [66]. In 2005, clinic trial had firstly reported that the successful control of posttransplant autoimmune hepatitis (AIH) with RAPA is proved by decreased transaminases (ALT and AST), immunoglobulin G, presence of autoantibodies, and histologic changes consistent with AIH on liver biopsy [67]. In addition, Rapamycin (RAPA) significantly ameliorated apoptosis via inhibiting mTORC2, PI3K, caspase-3, caspase-9 expression, and reactive oxygen species (ROS) generation, increasing ratio of Bcl2/Bax mRNA to attenuate tissues injury [16, 17, 68, 69]. In brief, Rapamycin may also have a pleiotropic and probable function in the hepatocyte protection and antihepatocyte apoptosis.

In our present study, after therapeutic and prophylactic administration of EZP, TSC expression was elevated in liver tissues induced by 2-AAF/PHx to suppress Rheb and mTOR activation, then leading to inactivation of TSC/mTOR signaling pathway. TSC/mTOR signals was inhibited to induce cascaded inhibitory reaction of hepatocyte apoptosis including lower-expression of Bax, Notch1, p70S6K and 4E-EIF and upregulated levels of Bcl-2 and Cyclin D. Expressions of these apoptosis-regulated proteins were reduced to inhibit caspase-3 activation, furthermore preventing apoptotic cell death. Meanwhile, high expression of Cyclin D in liver tissues enhanced self-proliferation ability of hepatocyte. The results were favorable to restoring hepatocyte function and physiological structure of liver tissues. The above results were found in the 2-AAF/PHx-induced liver injury rats treated by Rapamycin, which were sufficiently similar to EZP. As one of classic and representative mTOR pathway inhibitors, Rapamycin effectively inhibited hepatocyte apoptosis and suppressed the activation of TSC/mTOR signaling pathway in the present study. So, we deduced that it is crucial and definite that Rapamycin inactivated the TSC/mTOR signaling pathway to treat 2-AAF/PHx-induced liver injury. According to the effect of Rapamycin, These indicated that EZP ameliorated 2-AAF/PH-induced hepatocyte apoptosis via inhibiting TSC/mTOR signaling pathway.

As a TCM, the effective component and target of action of EZP are still not clear in the treatment of liver injury because of numerous and complicated chemical components. However, two main components of EZP, oleanolic acid (OA) and salidroside (Sal), have protective effects on liver. Many previous studies have demonstrated that OA and Sal possessed hepatoprotective action against ethanol, CCl4, and acetaminophen-induced hepatic injury in rats [70, 71]. Liu et al. demonstrated that OA can inhibit lipid peroxidation and CYP2E1 expression [72], increase free radicals, restore the altered cellular permeability [73], activate nuclear factor (erythroid-derived 2) like 2 (Nrf2) [74, 75], and have anti-inflammatory [76, 77] and antioxidant effects to protect animals from liver injury. Sal could protect liver tissue from injury by restoring hepatic glutathione peroxidase activities, inhibiting lipid peroxidation, decreasing MDA levels, and reducing the necrotic regions and expression of caspase-3 or hypoxia-inducible factor 1a in liver tissue [26, 78]. Sal or OA could suppress apoptosis via their antioxidant properties; mammalian target of rapamycin (mTOR), nuclear factor-*κ*B (NF-*κ*B), Jun N-terminal kinase (JNK), and mitogen-activated protein kinase (MAPK) signaling pathways; inhibition of excessive Ca^2+^ influx; and so on [79–83].

Although the aforementioned evidences are not enough to completely explain the mechanism of hepatoprotective action of EZP, they can provide the basis for the preventive and therapeutics effect of EZP on liver injury induced by 2-AAF treatment combined with PH. Future studies should explore the effective constituent of EZP protected liver tissues via regulating TSC/mTOR signaling pathway.

In conclusion, all results had indicated that hepatoprotective effect of EZP was possibly realized via inhibiting TSC/mTOR signaling pathway to suppress excessive apoptosis of hepatocyte.

## Figures and Tables

**Table 1:** Characterization of the herbs included in EZP.

Herbs	Percentage content (%)	Identified compounds	Effects	References
*Fructus Ligustri Lucidi (Nv Zheng Zi)*	50%	Oleanolic acid; ursolic acid; salidroside	Antioxidant; anticancer; promoted cell proliferation; antiaging; liver protection; immunoregulation	[20–25]

*Herba Ecliptae* *(Han Mo Lian)*	50%	Oleanolic acid; eclalbasa ponins	Liver protection; antioxidant; anti-inflammatory	[26–28]
